# Impact of therapeutic inhibition of oncogenic cell signaling tyrosine kinase on cell metabolism: in vivo-detectable metabolic biomarkers of inhibition

**DOI:** 10.1186/s12967-024-05371-9

**Published:** 2024-07-04

**Authors:** Kavindra Nath, Pradeep K. Gupta, Johnvesly Basappa, Shengchun Wang, Neil Sen, Cosimo Lobello, Jyoti S. Tomar, Alexander A. Shestov, Stepan Orlovskiy, Fernando Arias-Mendoza, Hilka Rauert-Wunderlich, David S. Nelson, Jerry D. Glickson, Mariusz A. Wasik

**Affiliations:** 1 Department of Radiology, University of Pennsylvania, 423 Curie Blvd, Philadelphia, PA 19104-6069 USA; 2 Department of Pathology, Fox Chase Cancer Center, 333 Cottman Ave, Philadelphia, PA 19111-2497 USA; 3 Advanced Imaging Research, Inc., Cleveland, OH USA; 4 Institute of Pathology, University of Würzburg, Würzburg, Germany

**Keywords:** Bruton’s tyrosine kinase (BTK), Magnetic resonance spectroscopy (MRS), Proton magnetic resonance spectroscopy (^1^H MRS), Tricarboxylic or Citric acid cycle (TCA), Signaling inhibition, Mantle cell lymphoma (MCL), Ibrutinib (IBR), ACP-196 (acalabrutinib), RNA Sequence analysis (RNA-Seq)

## Abstract

**Background:**

Inhibition of kinases is the ever-expanding therapeutic approach to various types of cancer. Typically, assessment of the treatment response is accomplished by standard, volumetric imaging procedures, performed weeks to months after the onset of treatment, given the predominantly cytostatic nature of the kinase inhibitors, at least when used as single agents. Therefore, there is a great clinical need to develop new monitoring approaches to detect the response to kinase inhibition much more promptly. Noninvasive ^1^H magnetic resonance spectroscopy (MRS) can measure in vitro and in vivo concentration of key metabolites which may potentially serve as biomarkers of response to kinase inhibition.

**Methods:**

We employed mantle cell lymphoma (MCL) cell lines demonstrating markedly diverse sensitivity of inhibition of Bruton’s tyrosine kinase (BTK) regarding their growth and studied in-depth effects of the inhibition on various aspects of cell metabolism including metabolite synthesis using metabolomics, glucose and oxidative metabolism by Seahorse XF technology, and concentration of index metabolites lactate, alanine, total choline and taurine by ^1^H MRS.

**Results:**

Effective BTK inhibition profoundly suppressed key cell metabolic pathways, foremost pyrimidine and purine synthesis, the citrate (TCA) cycle, glycolysis, and pyruvate and glutamine/alanine metabolism. It also inhibited glycolysis and amino acid-related oxidative metabolism. Finally, it profoundly and quickly decreased concentration of lactate (a product of mainly glycolysis) and alanine (an indicator of amino acid metabolism) and, less universally total choline both in vitro and in vivo, in the MCL xenotransplant model. The decrease correlated directly with the degree of inhibition of lymphoma cell expansion and tumor growth.

**Conclusions:**

Our results indicate that BTK inhibition exerts a broad and profound suppressive effect on cell metabolism and that the affected index metabolites such as lactate, alanine may serve as early, sensitive, and reliable biomarkers of inhibition in lymphoma patients detectable by noninvasive MRS-based imaging method. This kind of imaging-based detection may also be applicable to other kinase inhibitors, as well as diverse lymphoid and non-lymphoid malignancies.

**Supplementary Information:**

The online version contains supplementary material available at 10.1186/s12967-024-05371-9.

## Background

Small-molecule inhibitors that target cell signaling pathways are playing an ever-growing role in cancer treatment [1]. Therapy with Bruton’s tyrosine kinase (BTK) inhibitors such as ibrutinib (IBR) and ACP-196 (acalabrutinib) for lymphomas, including mantle cell lymphoma (MCL), is a prime example of such a targeted therapeutic approach, although drug resistance occurs in a large subset of patients, either as a primary event or over time [2–4]. Although the effects of inhibitors of BTK and other kinases on intracellular signaling pathways have been extensively studied, clinical evaluation of the activation status of these signaling pathways is currently not achievable.

Our previous in vitro studies [5] indicated that inhibition of BTK can be detected in MCL by measuring concentrations of the key metabolites lactate and alanine using noninvasive ^1^H-based magnetic resonance spectroscopy (^1^H MRS) imaging. In the current report, we have focused on in vivo studies in the mouse xenotransplant model to support the notion that this approach should become amenable to monitor response to inhibition of BTK and, prospectively, other kinase inhibitors in patients with lymphoma and, possibly, other malignancies. Furthermore, we have explored in-depth the impact of BTK inhibition on metabolic status of the MCL cells, foremost by performing metabolomic analysis. Here, we report that BTK inhibition profoundly affects cell metabolism both in vitro and in vivo. BTK inhibition induced widespread suppression of MCL cell metabolism, including decreases in the concentrations of lactate and alanine. Strikingly, significant decreases in lactate and alanine were detected in vivo as early as two and seven days after the initiation of BTK inhibitor therapy and directly corresponded with the suppression of MCL tumor growth. These findings not only demonstrate that BTK comprehensively controls cell metabolism but also indicate that changes in the concentration of kinase-regulated metabolites are highly sensitive and that early biomarkers of kinase inhibition are detectable both in vitro and in vivo. The implications of these findings are discussed.

## Materials and methods

### BTK inhibitors

IBR and ACP-196 were obtained from Selleckchem Chemicals and Janssen Pharmaceuticals, respectively. For in vitro experiments, IBR and ACP-196 were dissolved in DMSO. For in vivo studies, IBR was suspended in 2.2 ml of 10% hydroxypropyl B-cyclodextrin solution and administered orally to mice at a dose of 256 mg/kg.

### Cell lines and culture conditions

Two MCL patient-derived, low passage cell lines, MCL-RL and MCL-SL were developed in MA Wasik’s laboratory. MCL-RL cell line fully preserves the phenotype, mutational profile, and DNA methylation pattern of the parental MCL cells, as described previously [6]. The MCL-SL cell line, developed more recently from pleural effusion, also grew directly from the primary MCL cells without appreciable sub-selection and fully matches the phenotype and mutational profile of primary cells. JeKo-1 and REC-1 cell lines were obtained from the American Type Culture Collection (ATCC) and Maver cell line was a gift from L Wang, University of Chicago. All cells were cultured and authenticated as described [5, 7].

### Xenograft development

Animal studies were performed at the University of Pennsylvania Institutional Animal Care and Use Committee (IACUC). Ten million MCL cell lines were subcutaneously injected with Matrigel into male NSG mice obtained from the Stem Cell and Xenograft Core of the University of Pennsylvania. Tumor samples measuring 1 × 1 × 1 mm^3^ (5–10 pieces) suspended in Matrigel were subcutaneously implanted into male athymic nude mice obtained from the National Cancer Institute. All MRS studies were conducted on hemispherical tumors ~ 250  mm^3^ in volume, proven to be of sufficient size for optimal spectra, and fit the available coils.

### Cell count assay

MCL cells (2 × 10^4^ cells/well) were cultured in 96-well plates (Corning, Inc.) for 72 h with various concentrations of IBR. The cells were suspended in trypan blue–containing PBS and microscopically counted using a hemocytometer.

### Cell growth assay

Cells were plated in 96-well plates (3–5 × 10^3^ cells/well), treated with IBR or the drug vehicle, labeled with 3-(4,5-dimethylthiazol-2-yl)-2,5-diphenyltetrazolium bromide (MTT, Promega) at 5 mg/ml for 4 h, and solubilized with 10% SDS in 0.01 M HCl. The optical density (O.D.) of the culture supernatant, corresponding to the MTT conversion-mediated change in supernatant color, was determined at 570 nm using a Titertek Multiskan reader (Titertek Instruments).

### Cell cycle assay

BrdU/7-AAD staining was performed using the FITC BrdU Flow Kit (BD Pharmingen) according to the manufacturer's protocol. Briefly, the cells were incubated for 1 h with BrdU (10 μM), treated with DNase, exposed to fluorescent anti-BrdU, and stained for total DNA. The cells were assayed using a FACSCan flow cytometer (BD Biosciences). The data were analyzed using FlowJo v10.8.0 software.

### Apoptotic cell death assay

Annexin-V staining was performed using an annexin-V–FLUOS Staining Kit (Roche) according to the manufacturer's protocol. In brief, the cells were exposed to annexin-V-FLUOS and/or propidium iodide and analyzed using a Becton Dickinson FACScan flow cytometer and FlowJo v10.8.0 software.

### Analysis of glucose metabolism and mitochondrial respiration

MCL cell lines were tested by the Seahorse XFe96 analyzer (Agilent) using kits (Agilent) following the manufacturer’s instructions. Briefly, cells were seeded in 96-well plates at 1.2 × 10^5^ cells/well with medium supplemented with 2.0 mM glutamine for the glycolysis test or supplemented with 10.0 mM glucose, 1.0 mM sodium pyruvate, and 2.0 mM glutamine for the mitochondrial test. The extracellular acidification rate (EAR) was analyzed in glucose-free medium before and after sequential injections of 10.0 mM glucose, 1.0 µM oligomycin, and 50.0 mM 2-deoxy-D-glucose (2-DG), a glycolysis inhibitor. The oxygen consumption rate (OCR) was analyzed under basal conditions and sequentially after treatment with 1.0 µM oligomycin, 2.0 µM carbonyl cyanide 4-(trifluoromethoxy)phenylhydrazone) (FCCP), and 0.5 µM rotenone/antimycin A. The protein content of the cell lysates was measured by the Bradford assay and used to normalize the respiratory and glycolytic parameters. At least six technical replicates of the samples were analyzed, and the data were assessed using XF Wave Software (Seahorse Bioscience, Agilent).

### Lactate and glucose concentration analysis

The amounts of secreted lactate and glucose taken up by each MCL cell line were calculated using a YSI Glucose/Lactate Analyzer (YSI 2300 STAT Plus, YSI). MCL cells were added to fresh medium at 1–2 million cells/mL, treated for up to 68 h with vehicle or 500 nM IBR, and incubated for 4–-6 h before measurement.

### RNA Sequence (RNA-Seq) analysis

Variants were identified from the transcriptome using SAMtools, as previously described [8, 9], and expression calculations were performed using the Cufflinks package [10]. The expressed genes were identified using the Partek Genomic Suite and assigned to cell pathways and programs.

### Preparation of cell extracts for metabolomic and metabolic flux studies

The cells were treated for 24 h in triplicate with 500 nM IBR or vehicle. Polar metabolites were extracted using ice-cold 80% CH_3_OH extraction solution. For ^13^C tracing experiments, cells were treated with either 500 nM IBR or the drug vehicle for 48 h and incubated in fresh medium containing [1,6-^13^C2]-glucose and [U-^13^C5, U-^15^N2]-glutamine. For dynamic analysis, aliquots of cells were exposed to the labeled metabolic substrates for 20, 40, 60, 90, 160, or 180 min and examined by LC‒MS.

### Liquid chromatography (LC) and mass spectrometry (MS) of cell extracts

Metabolomic analyses were performed at the Proteomics & Metabolomics Facility of the Wistar Institute, as previously described [6]. Samples were analyzed by LC‒MS/MS on a Q Exactive HF-X mass spectrometer with a HESI II probe, in line with a Vanquish LC System from Thermo Fisher Scientific. Hydrophobic interaction LC separation (HILIC) was performed using a ZIC-pHILIC column (150 × 2.1 mm, 5.0 μM polymer) from EMD MilliporeSigma. Samples were analyzed using either full MS scans with polarity switching (all samples) or full MS/data-dependent MS/MS scans with separate acquisitions for positive and negative polarities (unlabeled samples in isotope tracing experiments; sample pool, QC, in nontracing experiments). The raw data were processed using Compound Discoverer 3.1 software (Thermo Fisher Scientific) with separate positive and negative polarity analyses. Pathway impact and enrichment analyses were performed using the MetaboAnalyst 5.0 software program at https://www.metaboanalyst.ca.

### Detection of metabolomic fluxes using ^13^C-labeled glucose and glutamine tracers

Metabolic fluxes were analyzed using the fragmented cumomer analysis (FCA) method, as recently described [11].

### In Vitro ^1^H MRS studies

MCL cells at ~ 15 million cells per sample were homogenized and sonicated twice, centrifuged at 16,000 × g for 10 min, lyophilized, and suspended in 600 µL of deuterium oxide (D_2_O) containing ~ 0.2 µM trimethylsilylpropanoic acid (TSP). High-resolution ^1^H MRS spectra were acquired on a 9.4 T/8.9 cm vertical bore MRSMRS spectrometer. A PRESAT pulse sequence (water suppression with presaturation pulses, Varian) was used with a 45° flip angle, repetition time (TR) = 8.8 s, sweep width (SW) = 6756.8 Hz, number of points (NP) = 16384, and number of averages (NT) = 128.

### In Vivo ^1^H MRS studies

In vivo MRS studies were performed on a 9.4 T/31 cm horizontal bore Bruker console. ^1^H MRS-detectable biomarkers were analyzed using a custom single-frequency (^1^H) slotted tube resonator. ^1^H MRS was performed on days 0, 2, and 7 after the first oral IBR administration (256 mg/kg). MCL-RL tumors were examined in male NSG mice, and REC-1, JeKo-1, and MCL-SL tumors were examined in male nude mice. A slice-selective double-frequency Hadamard Selective Multiple Quantum Coherence (Had-Sel-MQC) transfer pulse sequence was used to detect the lactate and alanine signals. The acquisition parameters were NP = 1000, TR = 4 s, and NT = 32. A localized water signal was also acquired using a similar slice without water suppression (TR = 4 s, NT = 4) to normalize the lactate and alanine signals [12, 13]. The stimulated echo acquisition mode (STEAM) pulse sequence was used to detect total choline (3.2 ppm) with the following parameters: NP = 2048, TR = 3 s, TE = 14 ms, and NT = 128. Voxels were chosen from a set of T1-weighted images to avoid lipid contamination from the surrounding skin. A localized water signal was also acquired using a similar voxel size and position without water suppression (TR = 3 s, TE = 14 ms, NT = 4) to normalize the choline signal [12]. NUTS and MestRec postprocessing software packages were used to process the in vivo MRS data. A 10 Hz exponential filter was used to improve the apparent signal-to-noise ratio of the ^1^H MRS data, and baseline correction was applied before plotting and calculating the peak areas [14].

### Tumor volume and body weight measurement

The tumor dimensions were measured using calipers in three orthogonal directions, and the volume was calculated using the equation V = π(a × b × c)/6, where a, b, and c are the length, width, and depth of the tumor, respectively. Body weights were measured using a balance.

### Histological and immunohistochemical MCL tumor tissue analysis

The tumor tissues were formalin-fixed and paraffin-embedded. Hematoxylin–eosin (H&E)-stained slides were generated for microscopic evaluation. Immunohistochemical staining of the MCL tumor tissues was performed as previously described [15]. In brief, the slides were heat-treated for antigen retrieval and incubated with standard primary antibodies from Dako to CD20 (B-cell marker), Cyclin D1 (CCND1; MCL marker), SOX-11 (MCL marker), and Ki-67 (proliferation index marker) to confirm the MCL phenotype and examine the cell proliferation rate. The stained slides were evaluated using light microscopy, and the images were acquired using a microscope attached to a camera (Leica).

### Statistical analysis

In each figure, the analyzed data are presented as the mean ± standard error of the mean (SEM). Two-tailed Student’s *t*-tests assuming variance homogeneity were used to calculate *p* values, and α = 0.05 was considered to indicate statistical significance. The fluxes determined from the metabolic network analysis are presented as the fitted flux ± standard deviation (SD) calculated using Monte-Carlo simulations. Gene expression *p-*values were corrected for multiple testing using the Benjamini–Hochberg method.

## Results

### Effect of BTK inhibition on the growth and survival of MCL cells

**Fig. 1:**
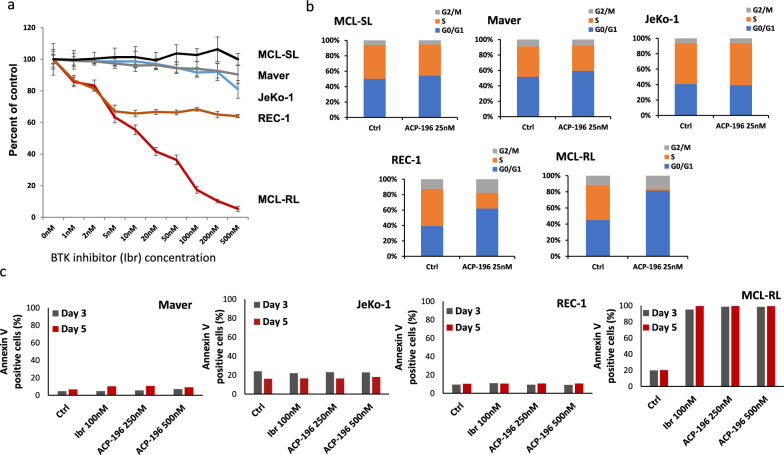
Effect of BTK inhibitors on growth and metabolism of MCL cells. **a** Results of MTT conversion assay with the depicted five MCL-patient derived cell lines after 72 h (3 day) incubation in vehicle (DMSO) or various concentrations of IBR ranging from 1 to 500 nmol/L. **b** Cell cycle distribution. **c** Cell apoptosis as detected by flow cytometry-detected Annexin V assay cell-surface expression after 3- and 5-day incubation with IBR or a second generation BTK inhibitor ACP-196, used at the depicted concentrations

Using the MTT conversion assay, we examined the effect of BTK inhibition on MCL cell growth at IBR concentrations ranging from 1 to 500 nM. Although the growth of MCL-RL cells was profoundly suppressed by IBR, the growth of REC-1 cells was moderately inhibited by the BTK inhibitor. JeKo-1 and Maver cells responded poorly to IRB, whereas MCL-SL cells were completely resistant to IRB. To confirm the diverse response of these MCL cell lines to BTK inhibition, we also evaluated its impact on cell cycle progression using the second-generation BTK inhibitor ACP-196 at a low dose of 25 nM (Fig. 1b and Supplemental Fig. S1). Although no effect of ACP-196 on the cell cycle was detected in MCL-SL, Maver, or JeKo-1 cells under these conditions, a marked decrease in the percentage of cells in S-phase was detected in the REC-1 cell population (40.7% for the control group *vs.* 16.8% for the treated group), and a profound decrease was detected in the MCL-RL cell population (34.2% *vs.* 1.8%), in agreement with the effect of IBR on cell growth (Fig. 1a). Regarding cell survival, IBR and ACP-196 induced massive apoptotic cell death only in MCL-RL cells (Fig. 1c and Figs. S2-S5), revealing further differences among the MCL cell populations in terms of their sensitivity to BTK inhibition.

### Impact of BTK inhibition on cell metabolism

**Fig. 2:**
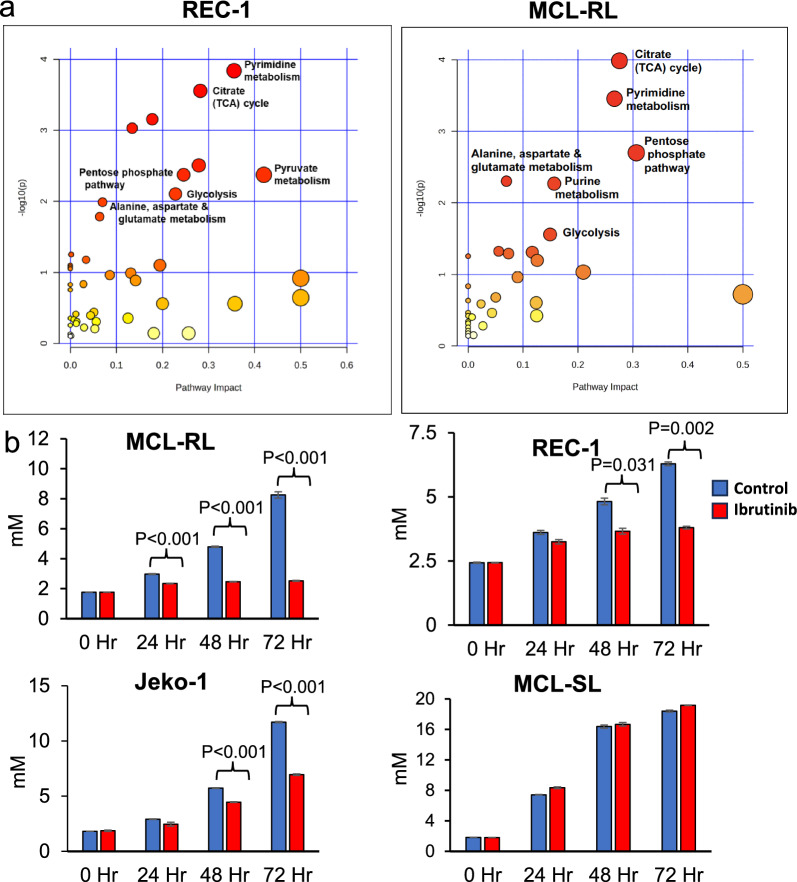
BTK inhibition-mediated suppression of metabolic pathways and extracellular lactate flux. **a** Impact of BTK inhibition on cell metabolism in sensitive MCL cells. The highlighted metabolic pathways affected in REC-1 and MCL-RL cells by their treatment with IBR have been identified by LC–MS and metabolome-targeting bioinformatics.** b** Impact of BTK inhibition in MCL cells on extracellular lactate. The depicted four MCL cell lines that differ in their sensitivity to BTK inhibition (Fig. 1a) were cultured with 500 nM of BTK inhibitor IBR and examined at the indicated time points for lactate concentration by using YSI 2300 biochemical analyzer. All experiments were performed in triplicates and the data are presented as mean ± SEM. with the *p*-values of statistical significance indicated

Next, we performed LC‒MS-based metabolomics using two cell lines with moderate (REC-1) and high (MCL-RL) IBR sensitivity. We performed untargeted steady-state polar metabolite profiling on these two cell lines and treated them with DMSO alone or with IBR-DMSO for 24 h. LC‒MS identified 166 and 185 polar metabolites in REC-1 and MCL-RL cells, respectively. Using a *p-*value < 0.05 and a fold change (FC) cutoff of ± 1.5, we selected 77 metabolites from REC-1 and 51 from MCL-RL for further pathway analysis. The IBR impaired a large spectrum of metabolic pathways in the two MCL cell populations, including pyrimidine and purine synthesis; the tricarboxylic acid (TCA) cycle; the pentose shunt; glycolysis; and the synthesis of the amino acids alanine, aspartate, and glutamate (Fig. 2a, and Figs. S6, and S7). Among the affected metabolic pathways, glycolysis/pyruvate metabolism/Warburg effect and alanine metabolism attracted our attention because we were able to detect the related metabolites lactate and alanine in intact cells using ^1^H MRS imaging [6]. Fig. S6 also shows the accumulation of choline, another metabolite detectable by in vivo ^1^H MRS as part of the total choline complex, which is typically dominated by phosphocholine [16].

Notably, the impairment of glycolysis and amino acid metabolism directly correlated with the BTK inhibition-mediated suppression of the mRNA expression of the key enzymes involved in these processes and the related oxidative phosphorylation, as observed in the REC-1 and MCL-RL cells (Supplemental Tables S1 and S2, respectively). The above findings indicate that BTK induces the expression of these metabolic enzymes and that the BTK inhibition-mediated decrease in the synthesis of lactate (a product of mainly glycolysis) and alanine (an indicator of amino acid metabolism) is due, at least in part, to the suppression of the expression of genes encoding enzymes involved in metabolism.

### Effect of BTK inhibition on lactate metabolism

To provide further evidence that BTK affects lactate metabolism, we analyzed the effect of IBR on the lactate concentration in MCL cells using a YSI 2300 biochemical analyzer. As shown in Fig. 2b, IBR suppressed lactate synthesis in MCL cell lines proportionately to inhibit their growth (Fig. 1a), with MCL-RL cells being the most affected and MCL-SL cells showing no decrease in lactate concentration. These findings were supported by the analysis of IBR-induced accumulation of intracellular glucose (Fig. S8), which remained unutilized for lactate synthesis. Accordingly, compared with IBR-untreated cells, MCL-RL-treated cells exhibited the greatest glucose accumulation, and MCL-SL did not.

### Impact of BTK inhibition on glucose and amino acid metabolism related to cell respiration

**Fig. 3:**
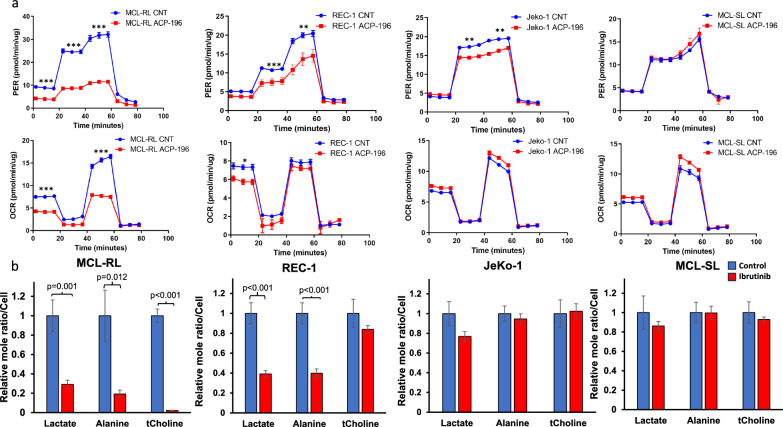
Metabolic impairment and ^1^H MRS-detectable biomarkers of BTK inhibition. **a** Effect of BTK inhibition on glucose metabolism and mitochondrial respiration. The index MCL cell lines were exposed for 48 h to 250 nM of BTK inhibitor ACP-196 or the drug vehicle and comprehensively tested for glucose metabolism (upper row) and mitochondrial respiration (lower row) by Seahorse-based examination. The depicted difference between BTK inhibitor-treated vs. control cells at the various stages of the tests were at least: *p < 0.05, **p < 0.001, and ***p < 0.0001. **b** Impact of BTK inhibition in MCL cells on lactate, alanine, and total choline percent change measured by high-resolution ^1^H MRS. All data are displayed as mean ± SEM, with the statistically significant *p*-values of the difference between BTK inhibitor-treated *vs*. controls also depicted

To gain deeper insight into BTK inhibition-induced changes in glucose metabolism in MCL cells, we performed Seahorse XF Glyco Stress Tests. In these assays, the basal proton efflux rate (PER) was determined, followed by a glucose pulse to increase glycolysis and subsequent treatment with oligomycin to determine the total glycolytic capacity. Finally, a pulse with 2-deoxy-D-glucose (2-DG) was used to evaluate the glycolytic reserve. The highly and moderately BTK inhibition-sensitive MCL-RL and REC-1 cells, respectively, displayed markedly impaired glucose utilization when treated with either ACP-196 (Fig. 3a, upper row) or IBR (Fig. S9, upper row). Conversely, the poor BTK inhibition-sensitive JeKo-1 cells showed a limited decrease in glucose utilization (Fig. 3a, upper row), whereas the resistant MCL-SL cell line did not show noticeable changes in glucose utilization (Fig. 3a and Fig. S9, upper rows). These findings further functionally link BTK with glycolysis and strongly support the notion that lactate, the end-product of glycolysis, is a highly reliable marker of BTK inhibition.

To evaluate the effect of BTK inhibition on oxygen consumption, which mainly reflects amino acid metabolism-driven mitochondrial respiration, we performed a Seahorse XF Mito Stress Test, in which basal respiration was assessed by inhibition, maximal respiration induction, and spare respiration capacity evaluation. The highly sensitive MCL-RL cells with BTK inhibition displayed a markedly diminished oxygen consumption rate (OCR) in response to BTK inhibition by either ACP-196 (Fig. 3a, lower row) or IBR (Fig. S9, lower row). In contrast, moderately (REC-1)- and poorly (JeKo-1)-sensitive and resistant (MCL-SL) cells displayed essentially no decrease or even a mild increase in the OCR in response to BTK inhibition (Fig. 3 lower row and Fig. S9), suggesting that the maintained amino acid-dependent mitochondrial respiration contributes to the limited sensitivity to BTK inhibition. Marked inhibition of both glycolytic and glutaminolysis flux by IBR in BTK inhibition-susceptible MCL-RL cells as well as of glycolytic flux but not glutaminolysis flux in poorly sensitive JeKo-1 cells (Fig. S10), further supporting this notion.

### ^1^H MRS imaging-based detection of BTK inhibition-mediated decreases in lactate, alanine, and total choline concentrations

Next, we analyzed the effects of IBR on lactate and alanine levels using ^1^H MRS-based imaging. We also examined the concentration of total choline, which is predominantly affected by phospho-choline [6], since the accumulation of choline as a substrate was observed via metabolomic analysis (Fig. S6), as well as our ability to detect total choline via ^1^H MRS (Fig. 3b).

IBR affected the lactate and alanine concentrations in MCL cells (Fig. 3b) proportionately to the degree of cell growth inhibition (Fig. 1a), with MCL-RL and REC-1 showing profound inhibition of lactate and alanine concentrations, JeKo-1 displaying a borderline effect for lactate and no visible effect for alanine, and MCL-SL showing no inhibition. Regarding total choline, MCL-RL cells displayed a profound IBR-mediated reduction, REC-1 and JeKo-1 showed a decreasing trend, while MCL-SL exhibited no change. In contrast to the changes in lactate, alanine, and total choline concentrations, we did not observe significant changes in taurine concentrations in any of these MCL cell populations (data not shown), confirming that BTK inhibition exerts a broad but not universal impact on tumor metabolism (Fig. 2a, and Figs. S6, and S7).

### In vivo detection of BTK inhibition-mediated decreases in lactate, alanine, and total choline concentrations by noninvasive ^1^H MRS and correlation with MCL tumor growth

**Fig. 4:**
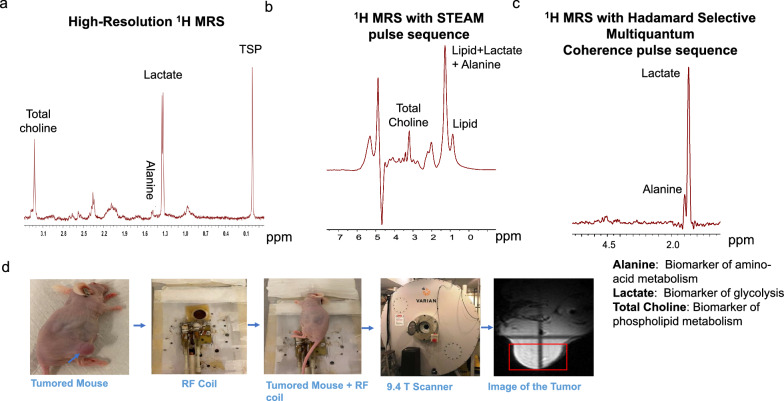
Representative in vitro and in vivo ^1^H MR spectrum. **a** Representative High-Resolution ^1^H MRS of MCL cell extract measured at 9.4 T vertical bore Varian magnet. **b** Representative ^1^H MRS of subcutaneous MCL xenograft measured at 9.4 T horizontal bore Bruker console using STEAM pulse sequence. **c** Representative ^1^H MRS of subcutaneous MCL xenograft measured at 9.4 T horizontal bore Bruker console using^1^H MRS with Hadamard Selective Multiple Quantum Coherence pulse sequence. **d** Representation of animal preparation for in vivo ^1^H MRS

**Fig. 5:**
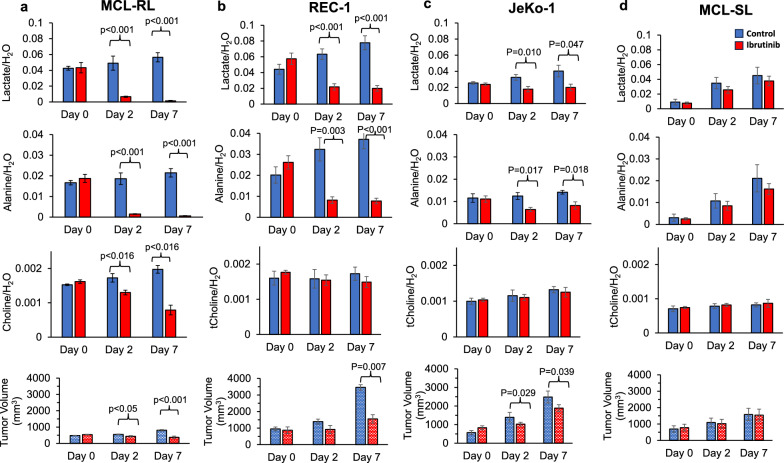
In vivo ^1^H MRS-detectable biomarkers of BTK inhibition-mediated suppression of tumor growth. The spectral peak area of lactate, alanine and choline signals normalized to the water signal measured by ^1^H MRS with Hadamard Selective Multiple Quantum Coherence (Had-Sel-MQC) transfer and stimulated echo acquisition mode (STEAM) pulse sequences, respectively, and tumor volume using calipers after treatment with IBR measured at Day 0, Day 2, and Day 7 in **a** MCL-RL (n = 5), **b** REC-1 (n = 5), **c** JeKo-1 (n = 5) and **d** MCL-SL (n = 5). IBR was given with the dose of 256 mg/kg, orally, once daily each day, thereafter. We have included drug vehicle treated controls in each MCL PDXs except MCL-RL. We have included drug vehicle treated controls in each of the four MCL xenotransplant models. The values are presented as the mean ± SEM, with the *p*-value of the difference between BTK inhibitor-treated vs. controls also depicted

We established an MCL xenotransplant mouse model suitable for the noninvasive study of lymphoma tumors by ^1^H-MRS imaging (Fig. 4), mimicking the peripheral lymphadenopathy observed in lymphoma patients. As shown in Fig. 5a, the intra-tumoral concentrations of the three index metabolites, in particular lactate and alanine, profoundly decreased in response to IBR therapy in the highly sensitive MCL-RL cells, recapitulating the in vitro results obtained with these cells (Fig. 3b). Notably, an associated decrease in the MCL-RL tumor volume was observed in response to IBR (Fig. 5a). A comprehensive analysis of the moderately sensitive REC-1 xenografts yielded similar results (Fig. 5b), demonstrating highly significant IBR-mediated inhibition of intratumoral lactate and alanine concentrations associated with the inhibition of tumor growth. In comparison, JeKo-1 tumors with low sensitivity to BTK inhibition yielded qualitatively similar results, although the changes in lactate, alanine, and tumor growth inhibition were much less robust and less statistically significant (Fig. 5c). The total choline concentration did not change noticeably in either REC-1 or JeKo-1 tumors (Fig. 5b and c, supporting the notion that lactate and alanine, rather than total choline, emerge as more universal biomarkers of BTK inhibition. Finally, resistant MCL-SL tumors displayed no detectable IBR-mediated inhibition of any of the metabolites or tumor growth (Fig. 5d). The effect of BTK inhibition on tumor growth was confirmed by immunostaining of resected tumors from IBR-treated *vs*. drug vehicle-treated mice for cell proliferation-associated Ki-67 protein (Fig. S11). Accordingly, the staining indicated a significant drug-induced decrease in the tumor cell proliferation (Fig. S11).

## Discussion

By employing MCL cell lines with diverse dependences on BTK activity, we found that effective BTK inhibition profoundly impaired cell metabolism. Although impact of BTK inhibition on cell metabolism has been noted previously by other investigators who observed induction of bioenergetic stress responses [17], our study revealed that the inhibition suppressed activity of the key metabolic pathways: foremost pyrimidine and purine synthesis, the citrate (TCA) cycle, glycolysis, and pyruvate and glutamine/alanine metabolism. This metabolic impairment has significant translational implications because imaging-based detection of decreases in tumor lactate (typically reflecting glycolysis) and alanine (an indicator of amino-acid-dependent oxidative metabolism) may serve as an early, reliable, and sensitive biomarker of the BTK inhibition response in patients with lymphoma.

Although the central role of BTK in B-cell receptor (BCR)-driven cell signaling is well established [3, 4, 18–21], our results demonstrate a novel and critical role of BTK in cell metabolism, given that BTK inhibition affects key metabolic pathways, including pyrimidine and purine synthesis, the TCA cycle, glycolysis, and pyruvate and alanine metabolism, in MCL cells (Fig. 2a). While the BTK inhibition-mediated impaired supply of DNA nucleotides affects the proliferation rate (Fig. 1a) and cell cycle progression (Fig. 1b) of MCL by directly preventing DNA synthesis, the other affected metabolic pathways are critical for generating energy to essentially support all key cell functions. These combined findings strongly suggest that the inhibition of cell metabolism is an important, if not critical, mechanism responsible for the efficacy of BTK inhibitors as therapeutic agents in lymphoma.

While the strong dependence of glycolysis on many cancers is well known and often referred to as the Warburg effect, the carcinogenic role of other metabolic pathways, such as glutaminolysis, has been less studied [22]. However, a single glutamine molecule produces up to 22.5 ATP molecules [23]; hence, even a limited degree of glutaminolysis can exert a crucial effect on tumor bioenergetics. Our noninvasive ^1^H-MRS measurement of alanine, a key player in the glutaminolytic pathway, can serve as a biomarker of glutaminolysis and its inhibition.

The impact of BTK inhibition on tumor metabolism reported here may have important translational implications for monitoring treatment response in patients with lymphoma and possibly other malignancies [3, 5–7, 16–18, 20]. Currently, there are no reliable methods for assessing the treatment effects of small-molecule kinase inhibitors in patients in a timely manner. At present, patients with MCL and other lymphomas are typically evaluated using F-fluorodeoxyglucose (FDG) positron emission tomography (PET)/computed tomography (CT) [24–26], and the therapeutic response is measured mainly by changes in tumor volume. However, inhibitors of BTK and other kinases are typically cytostatic rather than cytotoxic, leading to slow, often lasting significant tumor volume shrinkage. Furthermore, FDG PET/CT has not proven effective in predicting and identifying the response of lymphoma and other cancers to kinase inhibitors [24, 27], most likely because FDG PET measures the impact of inhibitors solely on glucose uptake [28] rather than a more global effect on tumor cell metabolism and growth. Other non-glycolytic metabolic pathways active in malignant cells, such as amino acid metabolism [16], seem to provide sufficient metabolic resources for cell survival and growth, leading to a lack of correlation between the FDG-PET results and actual tumor growth inhibition.

In this study, we identified lactate and alanine as biomarkers of early MCL response and resistance to BTK inhibition in vivo using a noninvasive 1H magnetic resonance (MRS) imaging method. Because ^1^H MRS can be broadly used in cancer patients [29], our study indicates that such radiologic image-based monitoring should prove effective in lymphoma patients treated with a BTK inhibitor and, by extension, in patients with other malignancies treated with analogous kinase inhibitors. The availability of such reliable and noninvasive detection methods is critical for lymphoma patients, given that only a subset of patients truly benefit from BTK-targeting therapy. This is particularly the case in MCL, where ~ 2/3 of MCL patients respond, while the remaining ~ 1/3 of patients do not respond to IBR [3, 30]. Furthermore, BTK inhibitor resistance also develops over time in a large subset of responding MCL patients [3–5, 30]. Even third-generation BTK inhibitors are effective in only ~ 50% of MCL patients [31, 32], further stressing the need for an effective method to promptly assess the early response to BTK inhibition. The same applies to kinase inhibitors used in malignancies other than lymphoma.

Although mutations in BTK and, less frequently, PLCG2 signaling downstream of BTK have been identified as responsible for resistance to BTK inhibitors [33] in some lymphoma cases, this mutational mechanism is rarely observed in MCL, indicating that other mechanisms, such as cell reprogramming [34], apparently involving metabolic rewiring from reliance on glycolysis to mitochondrial respiration (Fig. 3a) [35], are responsible for MCL resistance to BTK inhibition. These findings indicate again that the comprehensive examination of cell metabolism, as accomplished by ^1^H magnetic resonance (MRS) imaging, should prove effective in the detection of BTK inhibitor response and resistance in clinical settings, as we see in our in vivo preclinical MCL models (Fig. 5).

Using xenotransplant models of diffuse large B-cell lymphoma (DLBCL), our group has explored over the years MRS-detectable changes induced by multi-agent chemotherapy: CHOP [12], CHOP and rituximab (R-CHOP) immunochemotherapy [36], radiation therapy [37], and therapy with mTORC1 inhibitor [38]. These past studies have shown that lactate and, in some instances choline, may serve as biomarkers of response to the above therapies. Although important as a proof-of principle, these studies, however, have not translated into clinic because effects of CHOP ± R and radiotherapy can be reliably and relatively promptly detected by standard imaging measuring changes in the tumor volume and mTORC1 inhibitor has not so far been approved for therapy of DLBCL and other types of lymphoma. Our current comprehensive in vitro and in vivo and previous exploratory in vitro [6] studies to detect response of MCL cells and tumors to BTK inhibition have strong translational potential. Accordingly, our showing that not only lactate but also alanine can serve as early biomarkers of the inhibition may pave the way to a trial in lymphoma patients, given the clinical relevance of BTK inhibitors in MCL and other lymphomas, on one hand, and the late change in tumor volume induced by these inhibitors, on the other hand.

## Conclusion

In summary, our study provides insights into the dependence of cell metabolism on BTK, an emerging key component of BTK oncogenic activity. This study also showed that this BTK-driven regulation of cell metabolism has translational implications by identifying the index metabolites affected by BTK inhibition as reliable, sensitive, and early biomarkers of the effective inhibition of the kinase that are amenable to detection by noninvasive ^1^H MRS-based tumor imaging. Consequently, our study may contribute to a better understanding of malignant cell signaling and metabolism crosstalk and may impact the management of patients with MCL, as well as other lymphoid and nonlymphoid malignancies.

### Supplementary Information

Additional file 1.

## Data Availability

The datasets used and/or analyzed during the current study are available from the corresponding author upon reasonable request.

## Abbreviations

BTK: Bruton’s tyrosine kinase
MRS: Magnetic resonance spectroscopy
1H MRS: Proton magnetic resonance spectroscopy
TCA: Tricarboxylic or citric acid cycle
MCL: Mantle cell lymphoma
IBR: Ibrutinib
acalabrutinib: ACP-196
RNA-Seq: RNA sequence analysis
LC: Liquid chromatography
MS: Mass spectrometry
EAR: Extracellular acidification rate
2-DG: 2-Deoxy-D-glucose
OCR: Oxygen consumption rate
FCA: Fragmented cumomer analysis
D_2_O: Deuterium oxide
TSP: Trimethylsilylpropanoic acid
Had-Sel-MQC: Hadamard selective multiple quantum coherence
STEAM: Stimulated echo acquisition mode
PER: Proton efflux rate
BCR: B-cell receptor
FDG: F-fluorodeoxyglucose uptake
