# Supplementary material for: Impact of therapeutic inhibition of oncogenic cell signaling tyrosine kinase on cell metabolism: in vivo-detectable metabolic biomarkers of inhibition
Source: J Transl Med. 2024 Jul 4;22:622. doi: 10.1186/s12967-024-05371-9 (PMC11225145; doi:10.1186/s12967-024-05371-9)
Supplement: Supplementary file 1 — Additional file 1. [file 12967_2024_5371_MOESM1_ESM.pdf]

Table S1. REC-1 cells - IBR-induced changes in the expression of selected metabolic genes

Gene symbol	Enzyme/Protein name	Fold change	Up/Down-regulated	P value	KEGG Pathway/products affected
122622	Adenylosuccinate synthase 1	0.70644514	down	0.0095	Glycolysis / Gluconeogenesis
219	Aldehyde dehydrogenase 1 family member B1	0.652460806	down	0.0037	Glycolysis / Gluconeogenesis
226	Aldolase, fructose-bisphosphate A	0.533721354	down	0.0083	Glycolysis / Gluconeogenesis
514	ATP synthase F1 subunit epsilon	1.437045728	up	0.0042	Oxidative phosphorylation
517	ATP synthase membrane subunit c locus 2	1.290332969	up	0.0072	Oxidative phosphorylation
10632	ATP synthase membrane subunit g	1.498882211	up	0.0070	Oxidative phosphorylation
9114	ATPase H+ 2B transporting V0 subunit d1	1.695122449	up	0.0017	Oxidative phosphorylation
9550	ATPase H+2B transporting V1 subunit G1	1.4988822	up	0.015	Oxidative phosphorylation
2023	Enolase 1	0.403131401	down	0.0079	Glycolysis / Gluconeogenesis
2597	Glyceraldehyde-3-phosphate dehydrogenase	0.600603806	down	0.0052	Glycolysis / Gluconeogenesis
27165	Glutaminase 2	0.516419735	down	0.0094	Alanine, aspartate and glutamate metabolism
2752	Glutamate-ammonia ligase	0.645387081	down	0.0067	Alanine, aspartate and glutamate metabolism
2821	Glucose-6-phosphate isomerase	0.270679758	down	0.0022	Glycolysis / Gluconeogenesis
3939	Lactate dehydrogenase A	0.235987382	down	0.0053	Glycolysis / Gluconeogenesis
64077	Phospholysine phosphohistidine inorganic pyrophosphate phosphatase	1.759707649	up	0.0118	Oxidative phosphorylation
4707	NADH:ubiquinone oxidoreductase subunit B1	1.375139509	up	0.0078	Oxidative phosphorylation
5223	Phosphoglycerate mutase 1	0.503517817	down	0.0050	Glycolysis / Gluconeogenesis
441531	Phosphoglycerate mutase family member 4	0.534344467	down	0.0080	Glycolysis / Gluconeogenesis
5236	Phosphoglucomutase 1	0.721590567	down	0.0044	Glycolysis / Gluconeogenesis
5315	Pyruvate kinase M1/2	0.459483561	down	0.0025	Glycolysis / Gluconeogenesis
6391	Succinate dehydrogenase complex subunit C	1.421873621	up	0.0079	Oxidative phosphorylation
7381	Ubiquinol-cytochrome c reductase binding protein	1.323527713	up	0.0052	Oxidative phosphorylation

Note: Fold change = Treated/Control
$P_{adjusted}$ value < 0.05 is considered significant.
$P_{adjusted}$ value =P values corrected for multiple testing using the Benjamini and Hochberg method
Cells treated for 4 days

Table S2. MCL-RL cells - IBR-induced changes in the expression of selected metabolic genes

Gene name/symbol	Fold change*	Up/Down-regulated	P value#	KEGG Pathway affected
SLC2A1	1.35	up	0.000934	Glycolysis / Gluconeogenesis
SLC2A5	3.53	up	8.25E-96	Glycolysis / Gluconeogenesis
HK2	1.49	up	0.00306	Glycolysis / Gluconeogenesis
ACSS1	2.64	up	2.99E-36	Glycolysis / Gluconeogenesis
SLC16A1	-1.32	down	0.000162	Glycolysis / Gluconeogenesis
SLC16A6	-1.63	down	2.70E-09	Glycolysis / Gluconeogenesis
IDH3A	-1.41	down	1.06E-07	TCA cycle/Oxidative phosphorylation
GLS	-1.38	down	4.38E-06	Alanine, aspartate and glutamate metabolism
ACY3	1.26	up	0.000215	Alanine, aspartate and glutamate metabolism
GLUD1	-1.16	down	0.0457	Alanine, aspartate and glutamate metabolism
SLC38A5	-1.75	down	8.37E-17	Alanine, aspartate and glutamate metabolism
SLC1A5	-1.55	down	2.38E-12	Alanine, aspartate and glutamate metabolism
SDHA	1.28	up	0.00113	Oxidative phosphorylation
CAD	-1.55	down	3.75E-14	Alanine, aspartate and glutamate metabolism
ATP6V1C2	-1.38	down	2.55E-08	Oxidative phosphorylation
TCIRG1	1.89	up	1.91E-13	Oxidative phosphorylation

Note: Fold change = Treated/Control

$P_{adjusted}$ value < 0.05 is considered significant.

$P_{adjusted}$ value =P values corrected for multiple testing using the Benjamini and Hochberg method

Cells treated for 4 days

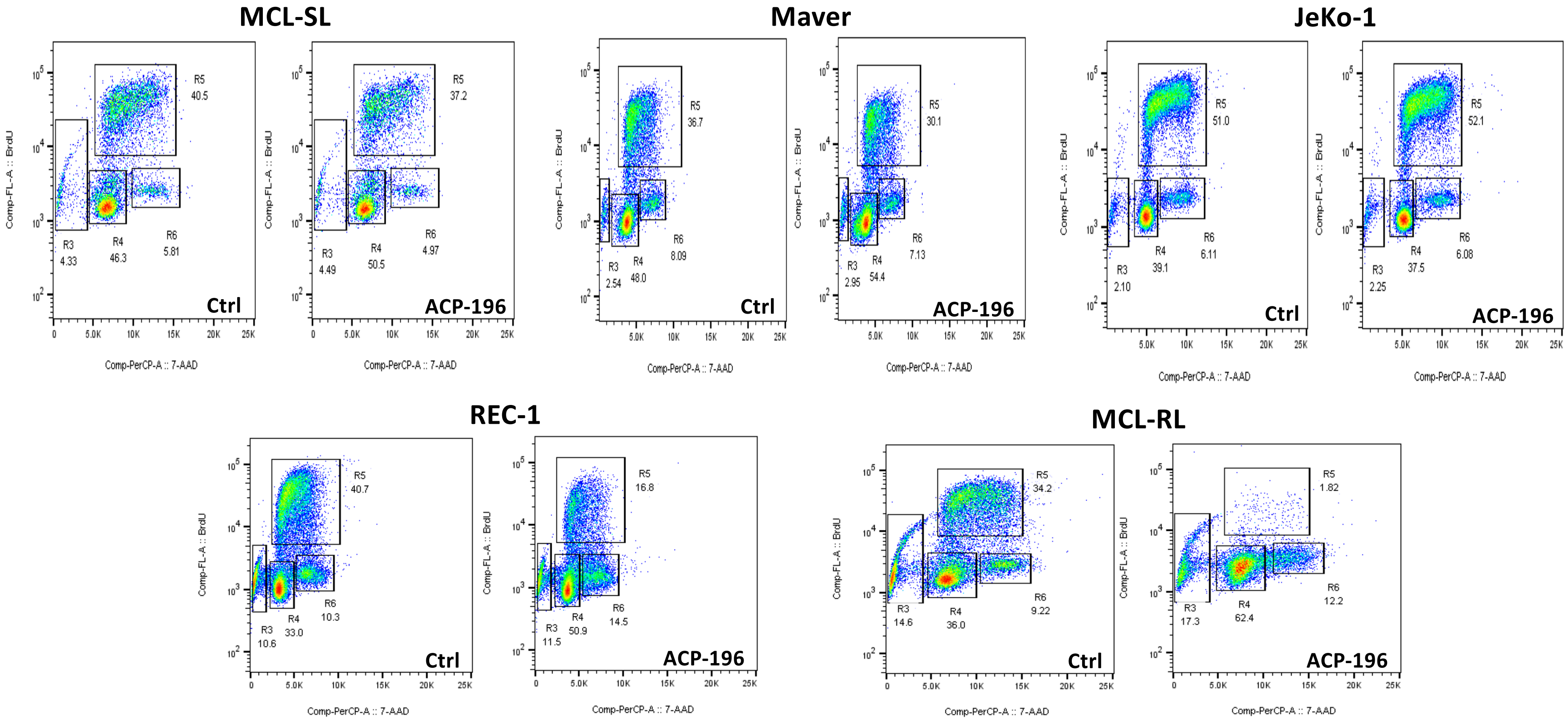

Figure S1. Inhibition of cell cycle progression in index MCL cells in response to BTK inhibition. The cell cycle was analyzed by flow cytometry in the depicted MCL cell lines after their co-culture for 48 hr with 25 nM of ACP-196; drug vehicle alone served as control. The depicted gates correspond to the following cell cycle stages: R3: sub-G0/G1; R4: G0/G1; R5: S phase; and R6: G2/M.

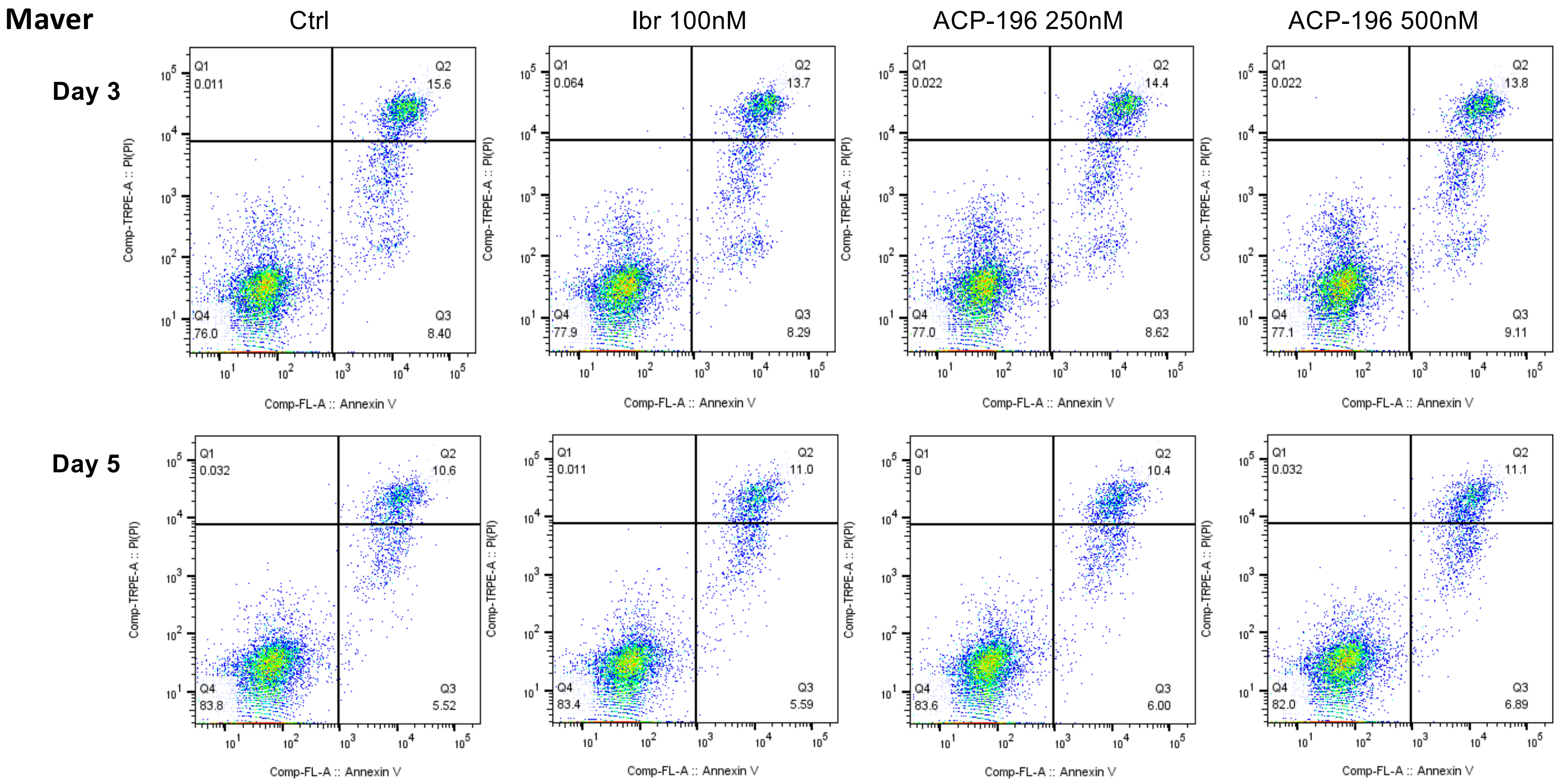

Figure S2. Lack of apoptotic cell-death induction by BTK inhibition in MCL Maver cells poorly responsive to the inhibition. The extent of apoptotic cell death was analyzed by flow cytometry using Annexin V antibody and propidium iodine (PI) staining in Maver cells after 48 hr co-culture with the either IBR or ACP-196 at the depicted doses; drug vehicle served as control.

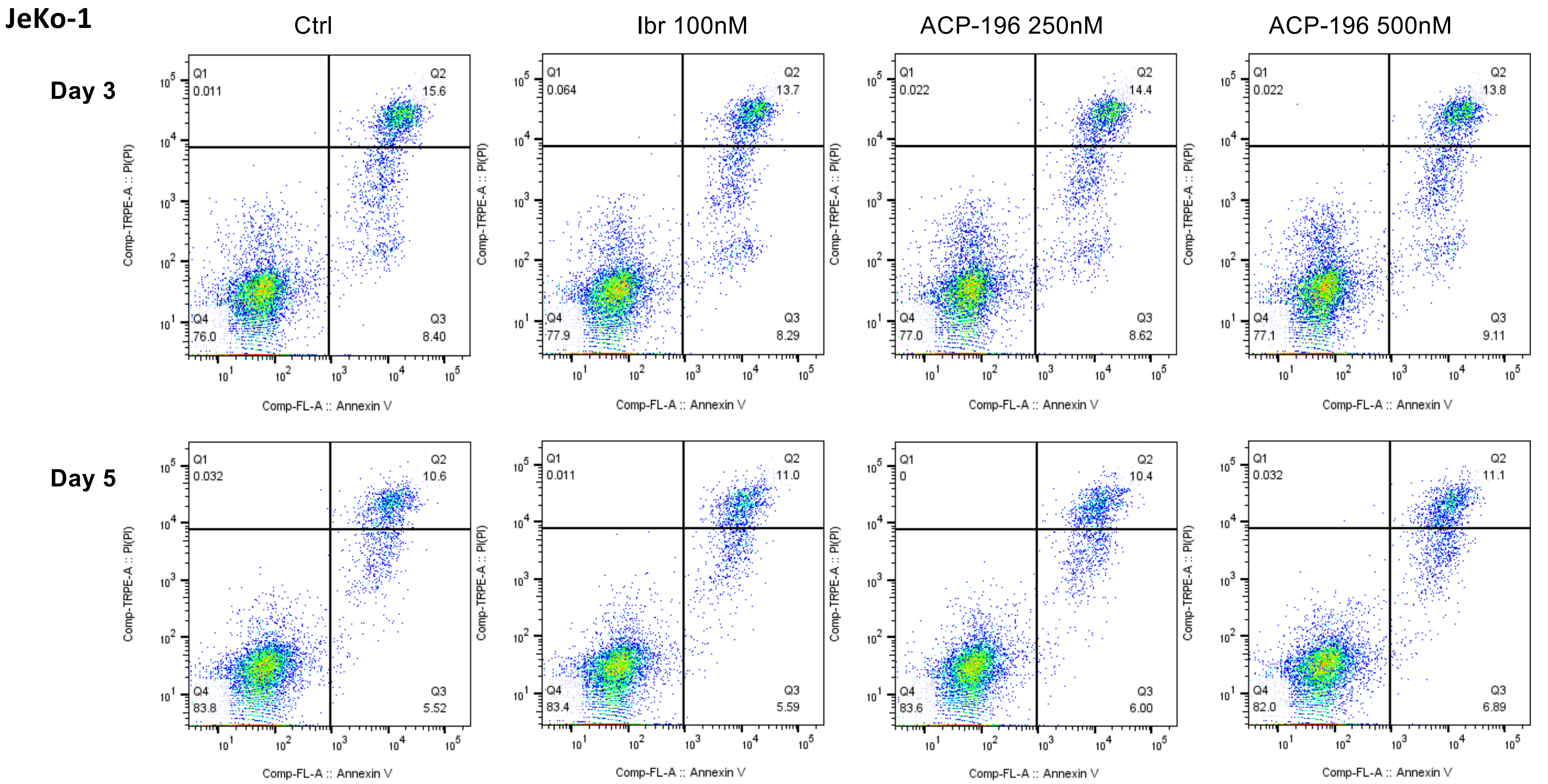

Figure S3. Lack of apoptotic cell-death induction by BTK inhibition in MCL JeKo-1 cells poorly responsive to the inhibition. Apoptotic cell death was analyzed by flow cytometry using Annexin V antibody and propidium iodine (PI) staining in Jeko-1 cells after their co-culture for 48 hr with the depicted BTK inhibitors or drug vehicle used as control.

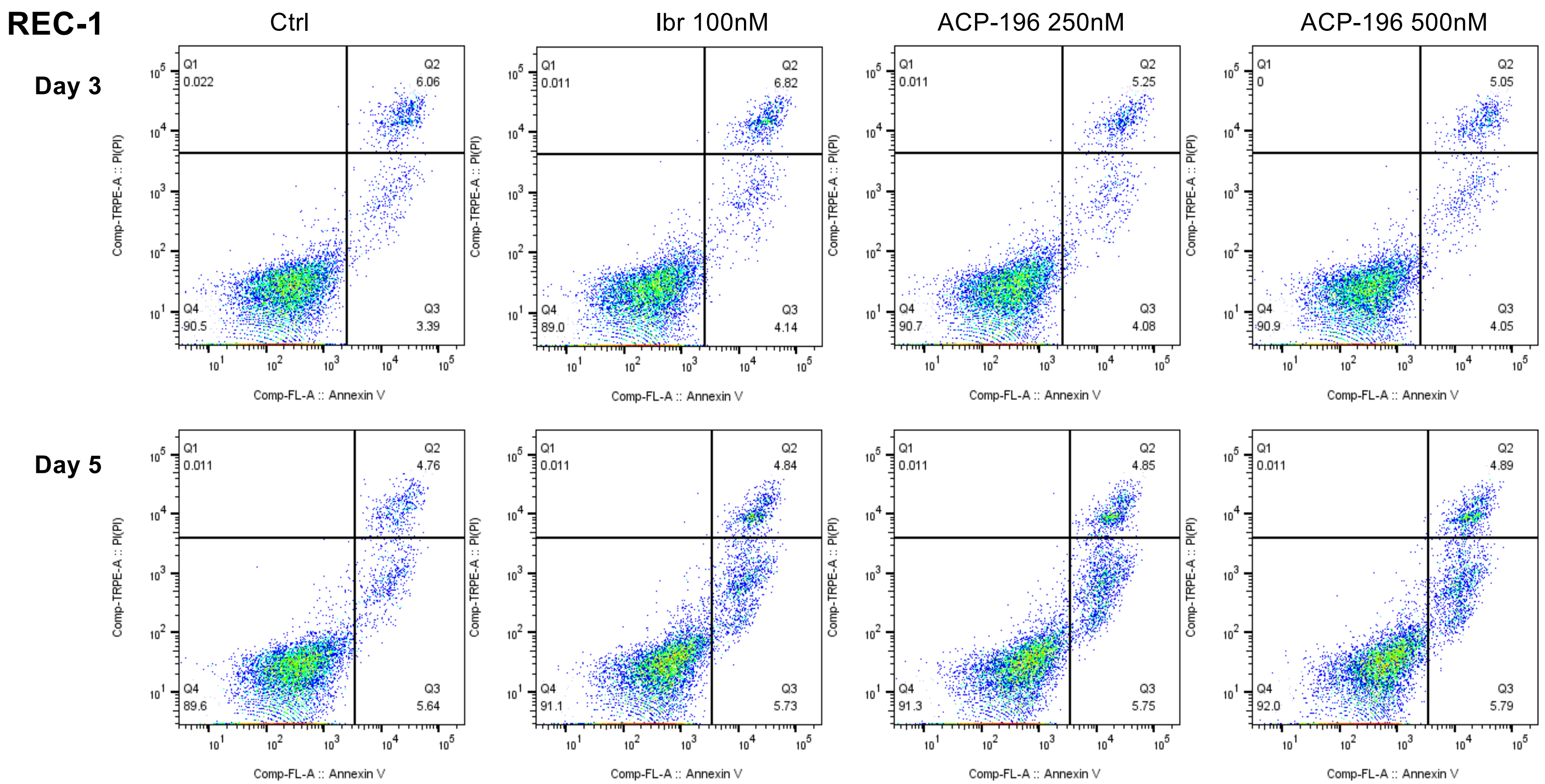

Figure S4. Lack of apoptotic cell-death induction by BTK inhibition in MCL REC-1 cells moderately responsive to the inhibition. The cell death was analyzed by flow cytometry using Annexin V antibody and propidium iodine (PI) staining in REC-1 cells after their co-culture for 48 hr with the BTK inhibitors or drug vehicle control.

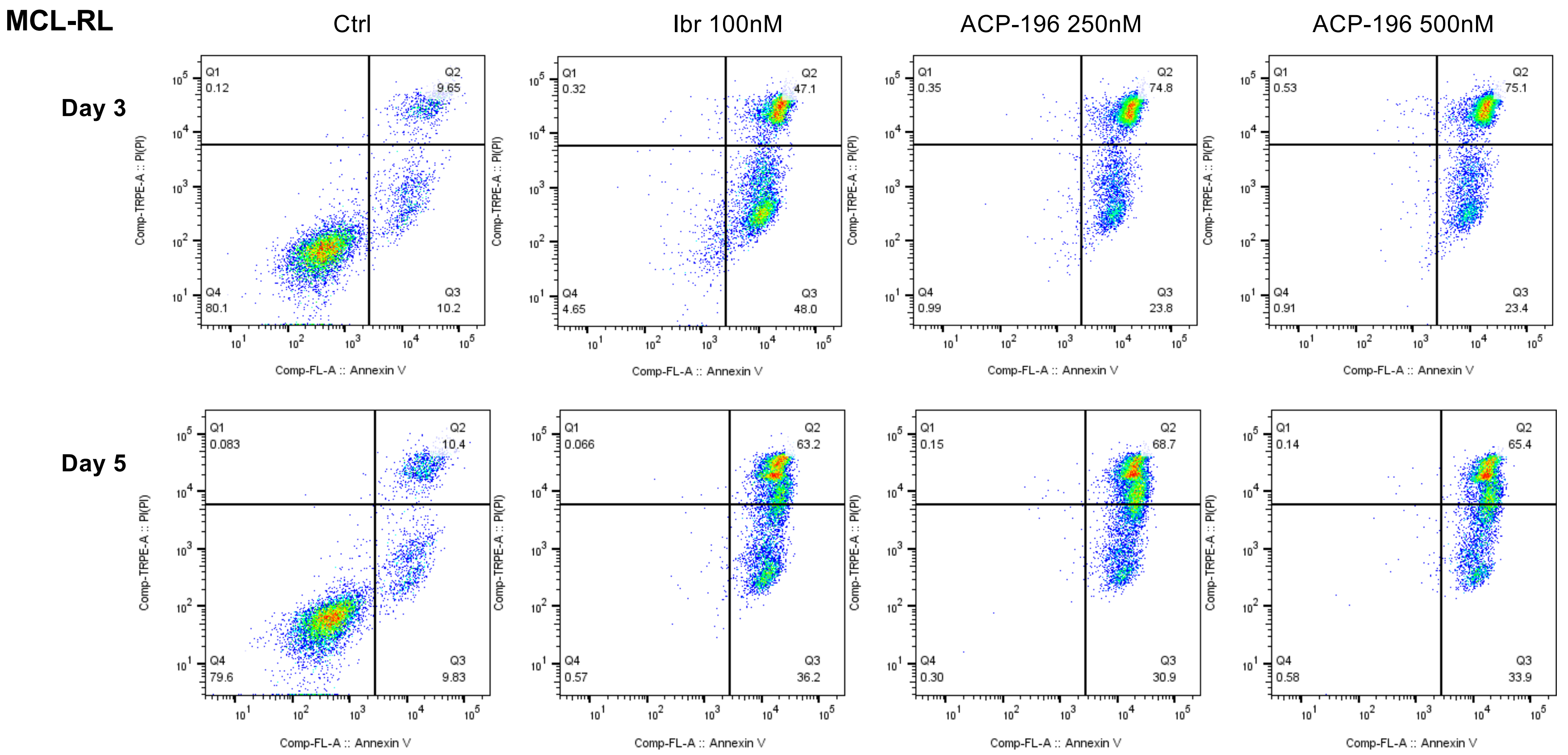

Figure S5. Induction of apoptotic cell-death by BTK inhibition in MCL-RL cells highly responsive to the inhibition. The cell death was analyzed by flow cytometry using Annexin V antibody and propidium iodine (PI) staining in MCL-RL cells after their co-culture for 48 hr with the BTK inhibitors or drug vehicle control.

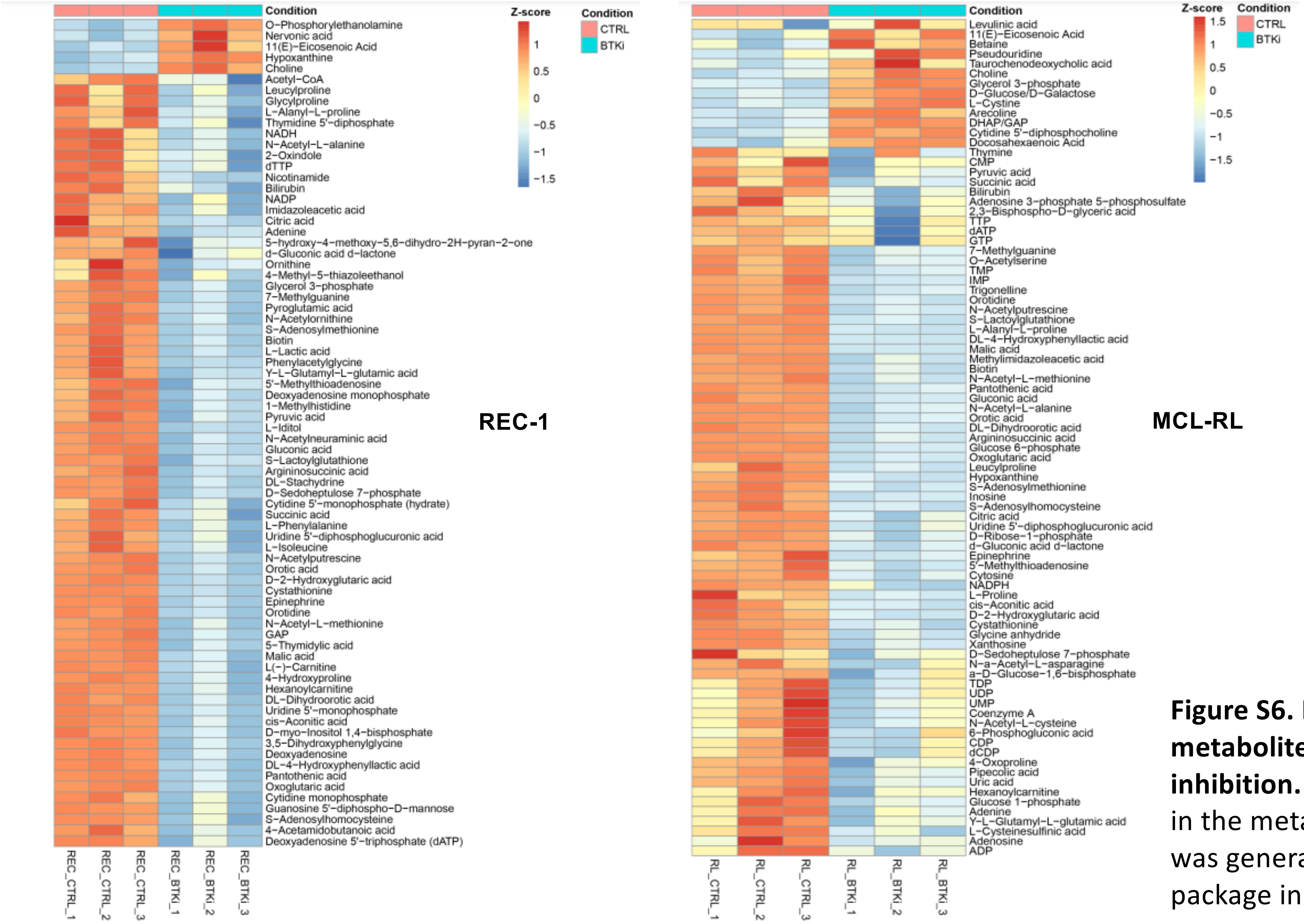

Figure S6. List of the identified metabolites affected by BTK inhibition. The heatmap of changes in the metabolite concentrations was generated using ggplot2 package in Rstudio (R version 4.0.2).

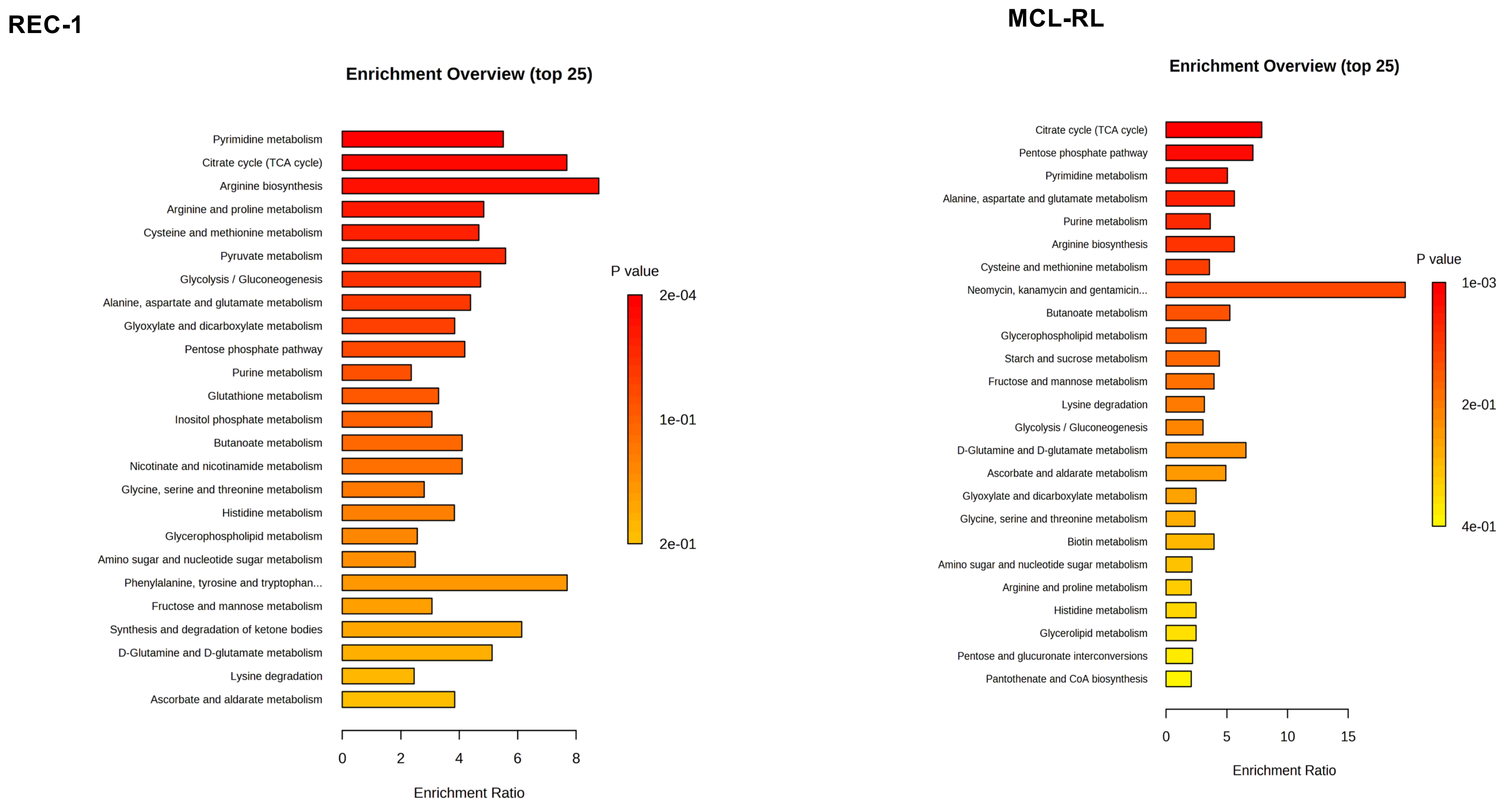

Figure S7. Key metabolic pathways affected by BTK inhibition. The metabolite enrichment analysis of significantly changed metabolites between the index IBR-treated and control MCL cell populations was performed using KEGG human metabolic pathways software program.

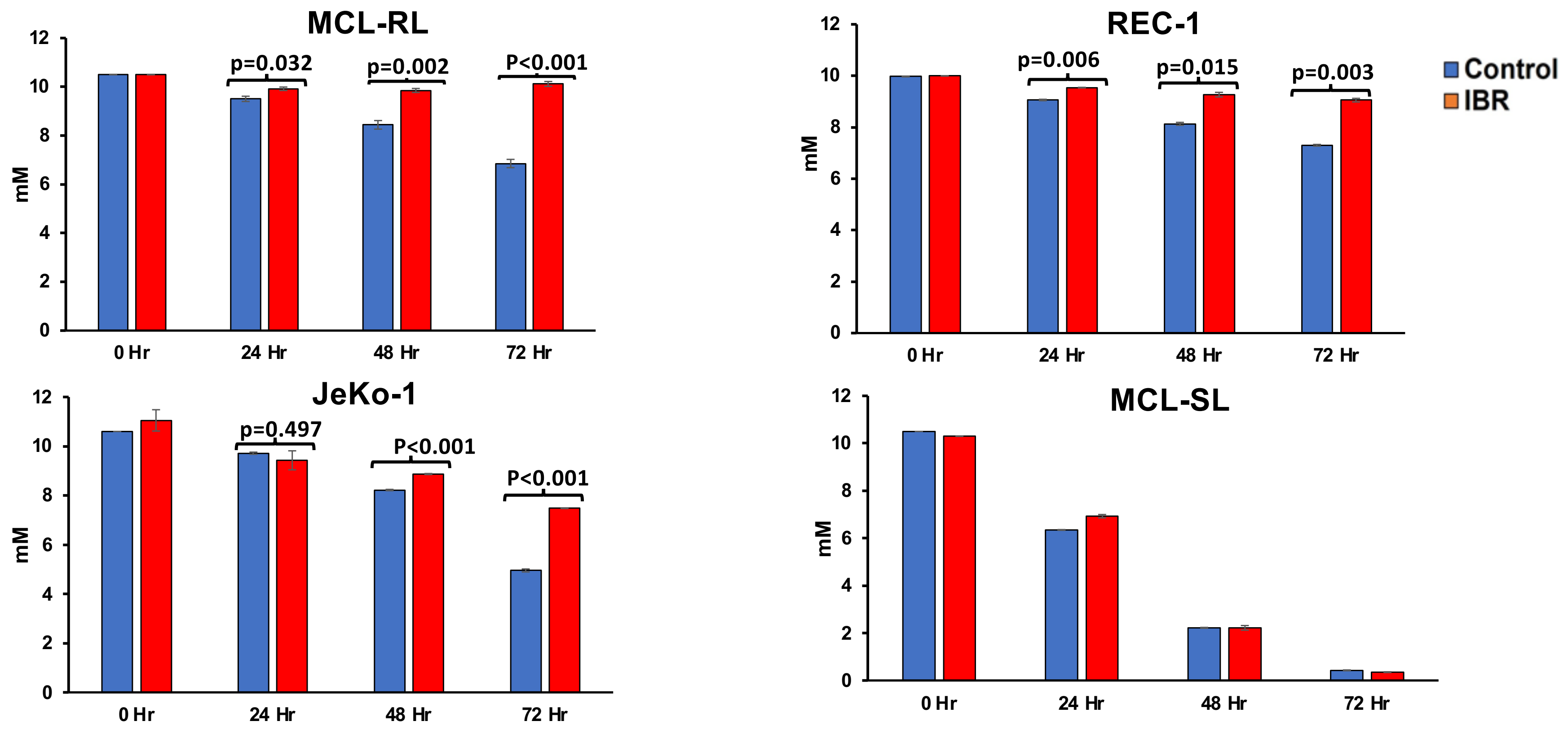

Figure S8. Impact of BTK inhibition in MCL cells on glucose concentration. The depicted MCL cell lines differing in their sensitivity to BTK inhibition (Fig. 1A) were cultured with 500 nM of BTK inhibitor IBR and examined at the indicated time points for concentration of intracellular glucose using YSI 2300 biochemical analyzer. All experiments were performed in triplicates and the data are presented as mean ± SEM with the p values indicated.

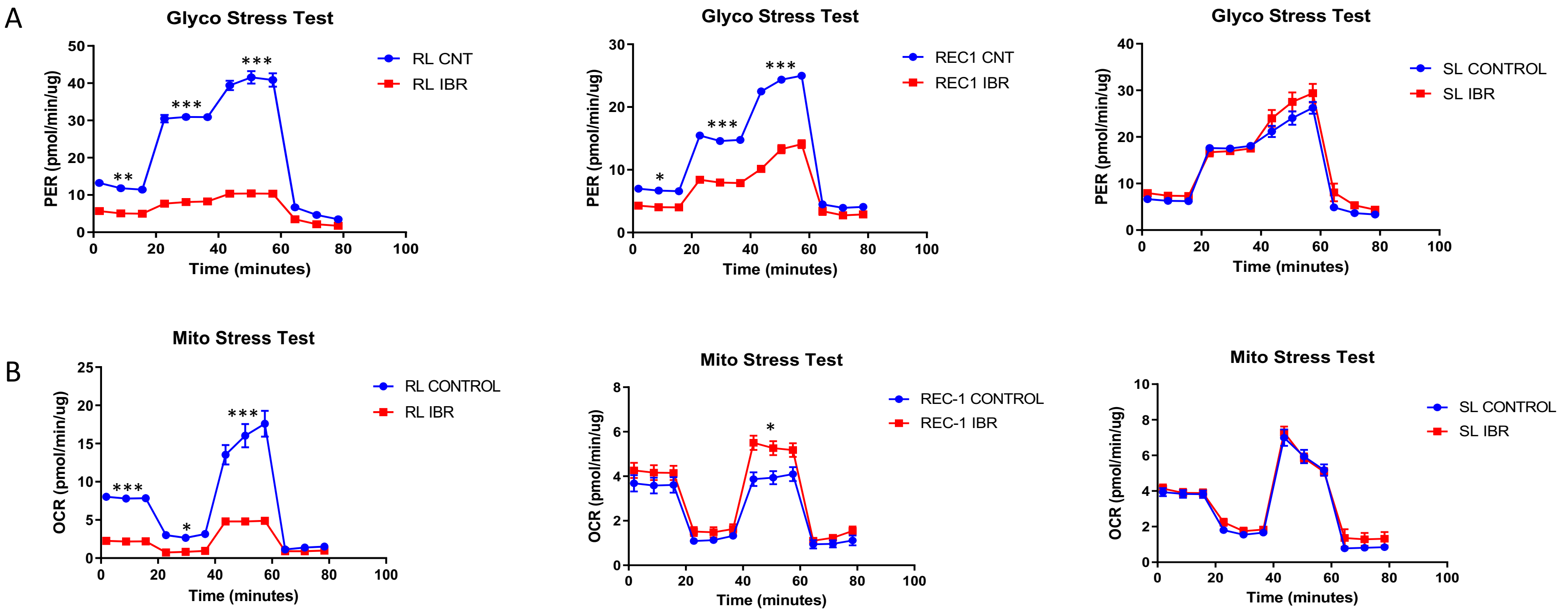

Figure S9. Effect of BTK inhibition on glucose metabolism and mitochondrial respiration. The depicted MCL cell lines were exposed for 48 hr to 200 nM of IBR or the drug vehicle and comprehensively tested for glucose metabolism (A) and mitochondrial respiration (B) by Seahorse-based examination. The depicted difference between BTK inhibitor-treated vs. control cells at the various stages of the tests were at least: * $p<0.05$, ** $p<0.001$, and *** $p<0.0001$.

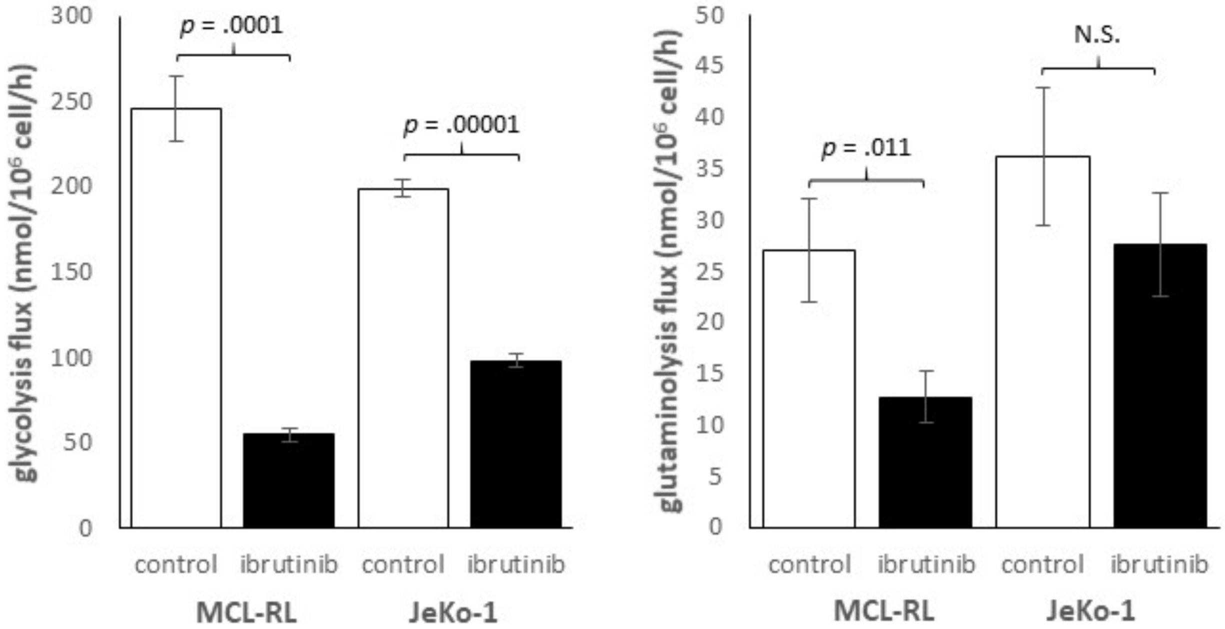

Figure S10. Glycolysis & glutaminolysis fluxes in MCL-RL vs. Jeko-1 cells. The cells were treated with 500 nM or drug vehicle for 48 hours, additionally exposed to the [1,6-13C2]-glucose and double-labeled [U-13C5, U-15N2] glutamine and studied for a dynamic labeling flow using 13C LC/MS coupled with Metabolic Flux Analysis (based on cumomers order 1).

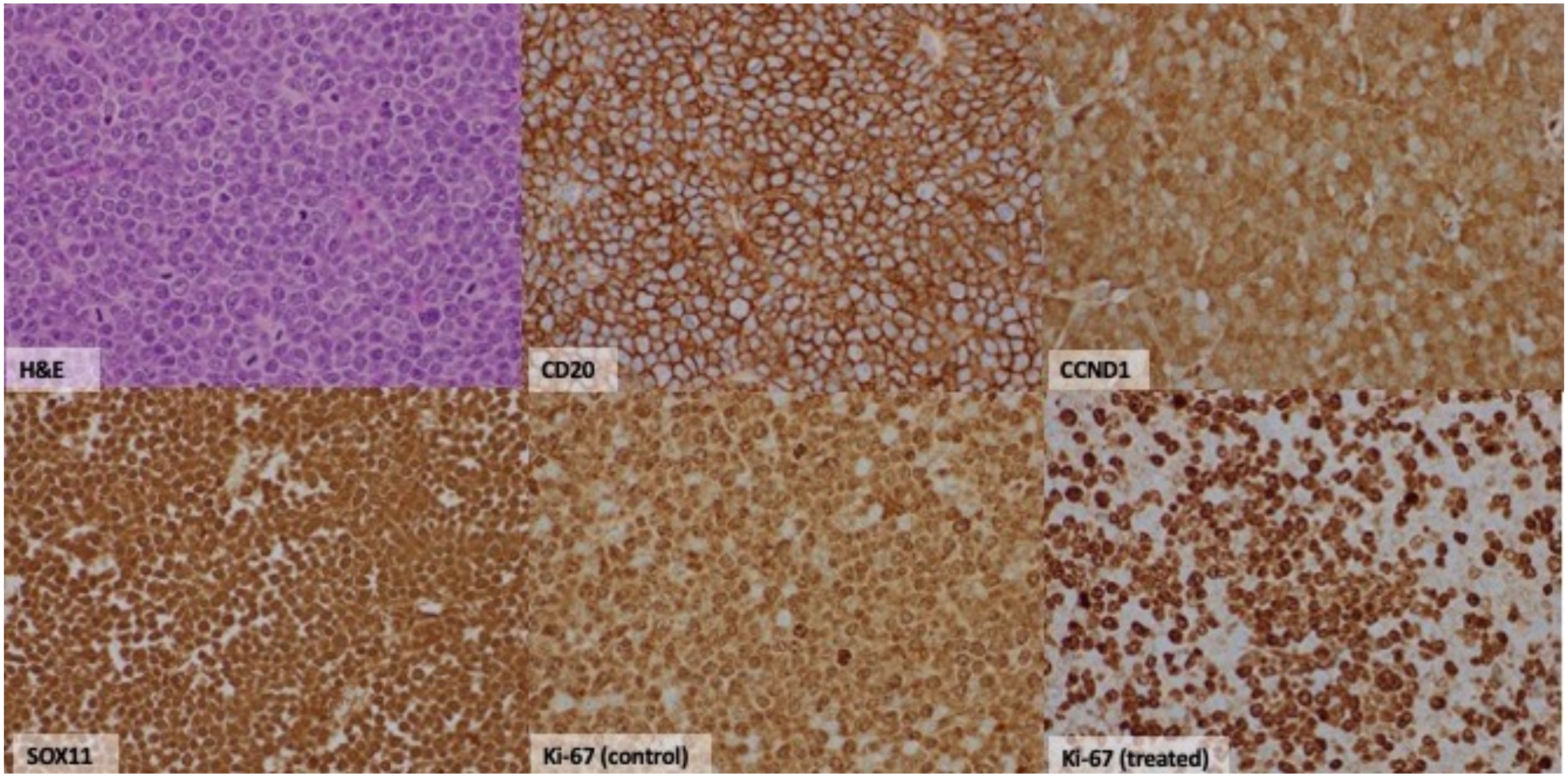

Figure S11. Phenotype and Ki-67 Expression in control vs. IBR-treated REC-1 Xenografts. Formalin-fixed, paraffin-embedded tumor tissues were stained with H&E to examine cell morphology, and anti-CD 20, -Cyclin D1 (CCND1), and -SOX11 antibodies to confirm human MCL phenotype of the tumors. The images shown are representative of seven different tumors (four from IBR-treated and three from control animals). The tumors were also stained for Ki-67 marker of cell proliferation index; depicted are Ki-67 stains of tumors from both control and IBR-treated mice (>95% vs. ~70% Ki-67+ cells, p value <0.0001).
